# Supplementary material for: The causal relationship between the human gut microbiota and pyogenic arthritis: a Mendelian randomization study
Source: Front Cell Infect Microbiol. 2024 Nov 26;14:1452480. doi: 10.3389/fcimb.2024.1452480 (PMC11629706; doi:10.3389/fcimb.2024.1452480)
Supplement: Supplementary file 1 [file DataSheet1.zip › mendelian test/ebi-a-GCST90027475.csv_forest.pdf]

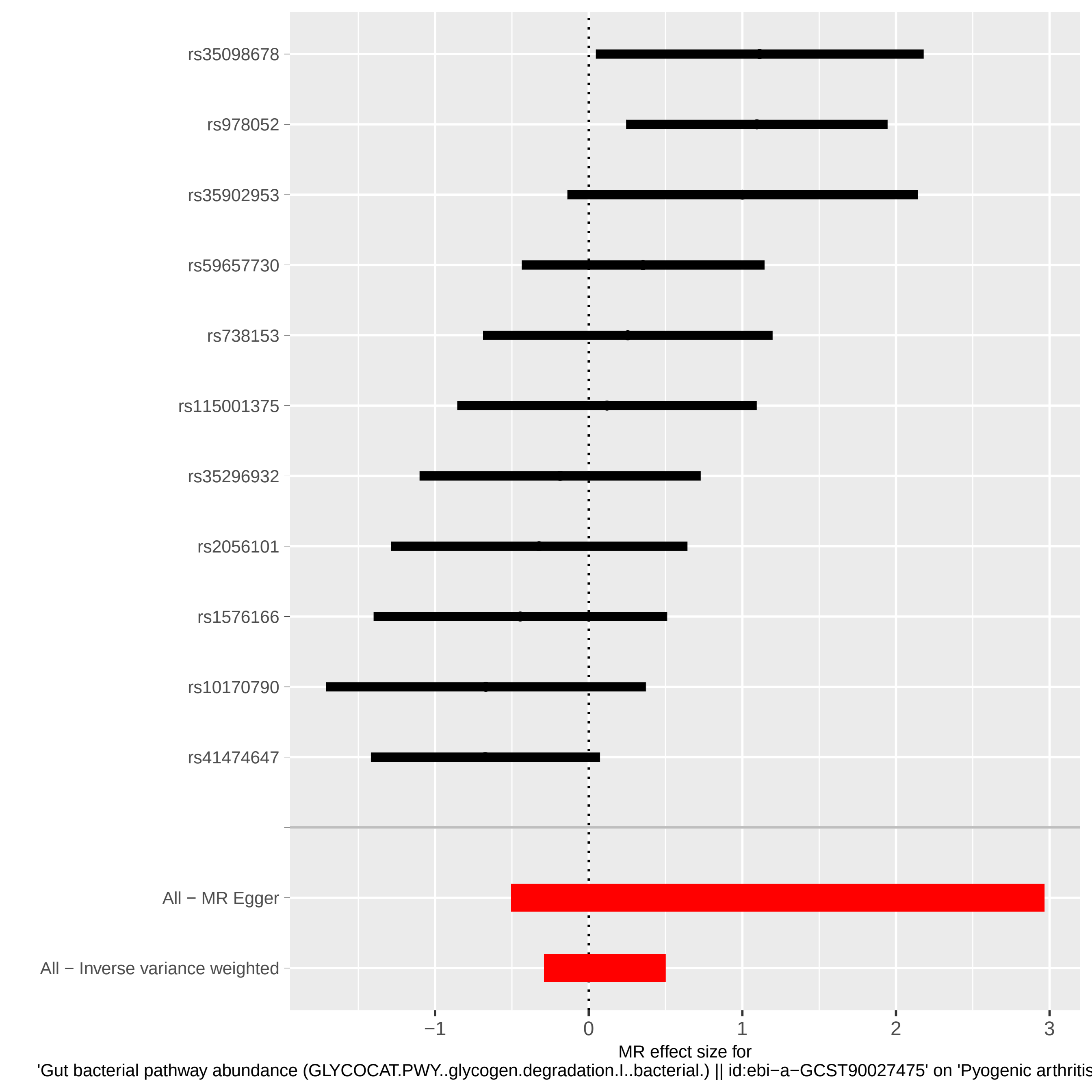
rs35098678
rs978052
rs35902953
rs59657730
rs738153
rs115001375
rs35296932
rs2056101
rs1576166
rs10170790
rs41474647
All − MR Egger
All − Inverse variance weighted
−1
0
1
2
3
MR effect size for
'Gut bacterial pathway abundance (GLYCOCAT.PWY..glycogen.degradation.I..bacterial.) || id:ebi−a−GCST90027475' on 'Pyogenic arthritis
